# Bioinspired Thermal Runaway Retardant Capsules for Improved Safety and Electrochemical Performance in Lithium‐Ion Batteries

**DOI:** 10.1002/advs.202103796

**Published:** 2021-12-19

**Authors:** Zhenhai Gao, Shun Rao, Tianyao Zhang, Fei Gao, Yang Xiao, Longfei Shali, Xiaoxu Wang, Yadan Zheng, Yiyuan Chen, Yuan Zong, Weifeng Li, Yupeng Chen

**Affiliations:** ^1^ State Key Laboratory of Automotive Simulation and Control Jilin University Changchun 130025 China; ^2^ Key Laboratory of Bio‐Inspired Smart Interfacial Science and Technology of Ministry of Education School of Chemistry Beihang University Beijing 100191 P. R. China

**Keywords:** capsules, fire, lithium‐ion batteries, retardants, thermal runaway

## Abstract

Vigorous development of electric vehicles is one way to achieve global carbon reduction goals. However, fires caused by thermal runaway of the power battery has seriously hindered large‐scale development. Adding thermal runaway retardants (TRRs) to electrolytes is an effective way to improve battery safety, but it often reduces electrochemical performance. Therefore, it is difficult to apply in practice. TRR encapsulation is inspired by the core–shell structures such as cells, seeds, eggs, and fruits in nature. In these natural products, the shell isolates the core from the outside, and has to break as needed to expose the core, such as in seed germination, chicken hatching, etc. Similarly, TRR encapsulation avoids direct contact between the TRR and the electrolyte, so it does not affect the electrochemical performance of the battery during normal operation. When lithium‐ion battery (LIB) thermal runaway occurs, the capsules release TRRs to slow down and even prevent further thermal runaway. This review aims to summarize the fundamentals of bioinspired TRR capsules and highlight recent key progress in LIBs with TRR capsules to improve LIB safety. It is anticipated that this review will inspire further improvement in battery safety, especially for emerging LIBs with high‐electrochemical performance.

## Graphical abstract

Using thermal runaway retardant (TRR) capsules can improve the lithium‐ion battery (LIB) safety without sacrificing electrochemical performance. This review aims to summarize the fundamentals of TRR capsules and highlight recent key progress in LIBs with TRR capsules to improve LIB safety. It is anticipated that this review will inspire further improvement in battery safety, especially for emerging LIBs with high‐electrochemical performance.

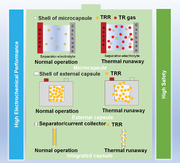

## 1 Introduction

Electric vehicles (EVs) have been paid much attention as a way to mitigate climate change.^[^ ^1^, ^2^, ^3^ ^]^ After many years of development, lithium‐ion batteries (LIBs) have become increasingly acceptable as the main power source of EVs, given their higher energy density and longer life cycle.^[^ ^4^, ^5^ ^]^ However, safety aspects have received increasing attention due to possible fire hazards, usually caused by the failure of on‐board large‐capacity power batteries in the form of thermal runaway (TR).^[^ ^6^, ^7^, ^8^ ^]^

Generally, TR occurs when the heat generated by exothermic reactions is not offset by the heat losses to the environment.^[^ ^6^, ^7^, ^8^ ^]^ This accumulated heat drives the temperature increase which, in turn, produces an exponential increase in the reaction rates. As the temperature of the battery rises to above ≈80 °C, the exothermic chemical reaction rate inside the batteries increases and further heats up the cell, resulting in a positive feedback cycle.^[^ ^9^, ^10^, ^11^ ^]^ The continuously rising temperatures may result in fires and explosions, especially for large battery packs.^[^ ^11^ ^]^ The TR process can be divided into three stages:^[^ ^9^, ^10^, ^11^ ^]^ the onset of overheating (stage 1), heat accumulation and gas release (stage 2), and combustion and explosion (stage 3).

Different material approaches to improving battery safety were summarized, solving problems corresponding to different TR stages.^[^ ^11^ ^]^ Reliable anode materials, multifunctional liquid electrolytes and separators, and overcharging protection can be used to solve the problems in stage 1 (i.e., the onset of overheating) during TR. Reliable cathode materials, thermally switchable current collector (CC), thermal shutdown separators, separators with high thermal stability, and battery packages with cooling function can be used to solve the problems in stage 2 (i.e., heat accumulation and gas release process) during TR. Flame‐retardant additives and nonflammable liquid electrolytes can be used to solve the problems in stage 3 (i.e., combustion and explosion) during TR.

As one of main material approaches to improving battery safety, considerable efforts have been contributed to the development of battery components with high thermal stabilities, such as the nonflammable liquid electrolytes, flame‐retardant electrolyte additives, fire‐resistant separators, fire‐resistant electrodes, and solid‐state electrolytes.^[^ ^12^, ^13^ ^]^ Li et al. have carried out a series of excellent research work on fire‐resistant materials for batteries.^[^ ^14^, ^15^, ^16^ ^]^ For examples, they report an ultrahigh‐capacity, fire‐resistant LiFePO_4_ (UCFR‐LFP)‐based nanocomposite cathode with fast electron and ion transporting behaviors, high mass loading of active materials, and excellent thermal stability.^[^ ^14^ ^]^ Such a unique UCFR‐LFP electrode offers a promising solution for next‐generation LIBs with high energy density, high safety, and wide operating temperature window.

Adding flame‐retardant additives to the electrolytes as described above is an effective method of mainly controlling the third stage of the TR process. Furthermore, it can control the second stage by improving the electrolyte thermal stability.^[^ ^17^, ^18^, ^19^, ^20^ ^]^ The commonly used flame‐retardant additives include phosphorus, triazine, ionic liquids, fluorine‐containing additives, bisphenol, etc.^[^ ^17^, ^18^, ^19^, ^20^ ^]^ Similar to flame‐retardant additives, another effective method for controlling TR in the first and second stages is to use electrolyte poisons, including benzylamine (BA), dibenzylamine (DBA), and trihexylamine (THA), diols, diamines, 1,1′‐(methylenedi‐4,1‐phenylene) bismaleimide (BMI), etc.^[^ ^21^, ^22^, ^23^, ^24^ ^]^

These flame retardants and poisoning agents prevent the occurrence or delay the development of TR. Therefore, both poisoning agents and flame retardants are referred to as TR retardants (TRRs) in this review. However, in most cases, huge amounts of TRRs are required to ensure a high level of cell safety, which consequently degrades the overall ionic conductivity of the electrolyte, resulting in poor electrochemical performance.^[^ ^11^, ^17^, ^18^, ^19^, ^20^, ^21^, ^22^, ^23^, ^24^ ^]^

Encapsulation can solve the problems faced by the above two methods by avoiding direct contact between the TRR and electrolyte, which is inspired by the functions of core–shell structures such as seeds, eggs, and fruits in nature.^[^ ^25^ ^]^ In these capsule structures, the shell isolates the core from the outside, so that the core and the outside do not affect each other. In addition, the shell has to break as needed to expose the core, such as in seed germination, chicken hatching, etc. Before the seed germinates, the seed coat (i.e., shell) has a protective effect on the embryo and endosperm (i.e., core) and isolates them from the outside. When the seed germinates, the seed coat ruptures and the seedling transformed from embryo and endosperm grows from the seed coat. Cells, seeds, eggs, fruits, etc., also have similar core–shell structures and functions. In summary, the main function of the shell is to protect and isolate the core from the outside, and releasing the core to the outside when needed. Similarly, TRR encapsulation avoids direct contact between the TRR and the electrolyte, so it does not affect the electrochemical performance of the battery during normal operation. When LIB thermal runaway occurs, the capsules release TRRs to slow down and even prevent further thermal runaway. Therefore, the electrochemical performance of the battery is hardly affected, and controllable release of the TRR can be achieved.

The application of capsules in LIBs has quietly increased in the field of battery safety in recent years, but it has not received the attention it deserves. In this review, we will define the capsule concept, classify capsule types and synthesis methods, summarize the electrochemical and safety performance of batteries with bioinspired TRR capsules, and discuss other applications of capsules in LIBs. This review provides a reference for battery design that considers both safety and electrochemical performance, thus providing an applicable solution to battery safety problems.

## 2 Concept of Lithium‐Ion Batteries with TRR Capsules

**Figure 1:**
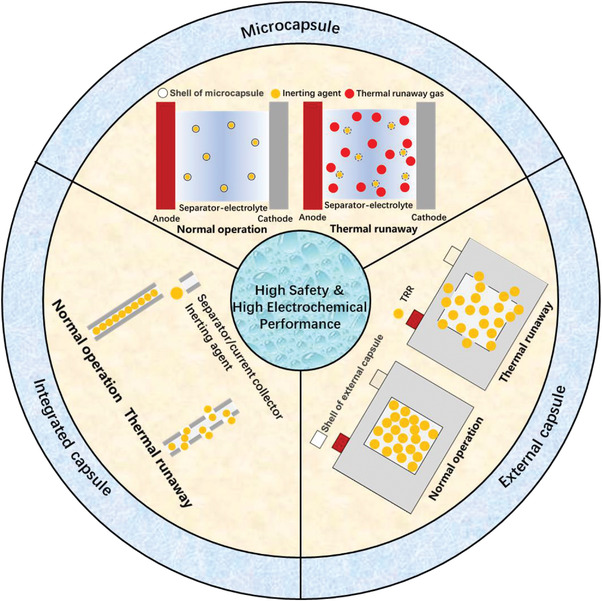
Concept of high‐safety and high‐electrochemical performance cells with TRR capsules.

Inspired by the isolation and protection functions of natural products with a core–shell structure, the encapsulation of TRRs has been developed to solve trade‐off relationships between safety and electrochemical performance in battery design. TRR capsules are filled with TRRs (i.e., the core) that reduce the reactivity of the cell materials when TR is occurring, and the shell of the capsule isolates the TRRs from the electrolyte when the cell is working normally, to prevent the TRR from deteriorating battery performance. When the cell experiences TR, the capsule releases the TRRs and dilutes the internal substances of the cell such as the electrolyte and TR gases, as shown in **Figure** **1**. This is very similar to the process of seed germination, chicken hatching, etc. In addition, when TR is occurring, the shell has function of sensitive materials to senses temperature, pressure and other factors, and responds through structural collapse, rupture, etc., to release the TRR dynamically to suppress TR. Therefore, this type of battery integrates the characteristics of smart technology and bionics. We defined LIBs with TRR capsules are a new type of battery, i.e., TRR capsule‐based batteries, in which the TRR capsules inside the cell automatically release when TR is occurring. It can also be called a thermal runaway self‐inhibiting battery, because the TRR capsule is part of the cell.

According to dimensions, shell materials, etc., capsules can be divided into microcapsules, external capsules, and integrated capsules, as shown in Figure 1. They are discussed in detail as follows.

Microcapsules generally refer to capsules with a size between 1 and 1000 µm.^[^ ^26^ ^]^ Preparation of microcapsules dates back to the 1950s when Green and Schleicher produced microencapsulated dyes by complex coacervation of gelatin and gum arabic for the manufacture of carbonless copying review.^[^ ^27^ ^]^ This technology aims to immobilize, protect, structure, and release the encapsulated substance according to a specific end use. Microencapsulation has been widely used in the pharmaceutical, food, agriculture, coatings, personal care, imaging, textiles, and other industries.^[^ ^28^, ^29^, ^30^, ^31^ ^]^ In batteries with microcapsules, the microcapsules are generally coated on the separator, dispersed in the electrolyte, or doped in the electrode material.^[^ ^32^, ^33^, ^34^, ^35^ ^]^

External capsules are similar to microcapsules, and the main difference is that they are larger. Their diameters are greater than 1000 µm.^[^ ^26^ ^]^ Therefore, in batteries with external capsules, TRRs are usually filled into the external capsules in the form of packages and then the external capsules are embedded between the separate‐based cell core and the cell housing.^[^ ^36^ ^]^

In batteries with integrated capsules, interior parts like the separator, CC, etc., are generally designed to be hollow and filled with TRRs.^[^ ^37^, ^38^ ^]^ By using ultralight materials, this type of battery can increase the battery's specific energy while improving battery safety.

It should be noted that due to the large size of external capsules, they cannot be coated on the separator, dispersed in the electrolyte, doped in the electrode material like microcapsules. In addition, they have their own independent shell, rather than using the cell interior parts as the shell in an integrated capsule. Therefore, external capsules are fundamentally different from the other two types of capsules.

**Table** **1** shows the core materials, shell materials, and positions of different types of capsules reported.^[^ ^32^, ^33^, ^34^, ^35^, ^36^, ^37^, ^38^ ^]^ Core materials, i.e., TRRs, include 1,1,1,2,2,3,4,5,5,5‐decafluoro‐3‐methoxy‐4‐(trifluoromethyl)‐pentane (DMTP), tris(2‐chloroethyl phosphate) (TCP), boehmites (AlOOH) with a large size (AlOOH‐L) and a small size (AlOOH‐S), paraffin wax (PW), triphenyl phosphate (TPP), 9,10‐dihydro‐9‐oxa‐10‐phosphaphenanthrene‐10‐oxide (DOPO), and pentadecane. Shell materials include polymethyl methacrylate (PMMA), poly(urea‐formaldehyde) (PUF), propargyl alcohol propoxylate (PAP), poly(vinylidene fluoride‐hexafluoropropylene) (PVDF‐HFP), polyacrylonitrile (PAN), copper (Cu), and aluminum (Al). For convenience, we use the convention core@shell to describe the capsules. The release of TRRs can be controlled by temperature, extrusion, etc. The release property of TRRs will be discussed in Section 5. It should be noted that the PW@PAN separator cannot be regarded as integrated capsules strictly because it lacks the release process of the core material.^[^ ^39^ ^]^ This review will also introduce this strategy and classify it as a kind of integrated capsule, due to that this strategy uses capsule strategy and has achieved improved safety and electrochemical performance of batteries.

**Table 1:** Materials and locations of different types of capsules

Type	Core	Shell	Location	Refs.	Release temperature [°C]
Microcapsule	DMTP	PMMA	Coated on a separator and mixed with electrolyte	[32]	≈127^a)^
	TCP	PUF	Mixed with electrolyte (LiClO_4_ EC/DMC and LiPF_6_ EC/EMC)	[33]	≈130^b)^
	AlOOH‐L	PUF	Mixed with cathode‐active material (LiFePO_4_)	[34]	–
	AlOOH‐S	PUF	Mixed with cathode‐active material (LiFePO_4_)	[34]	–
	TPP	PUF	Mixed with cathode‐active material (LiFePO_4_)	[35]	≈170^b)^
	DOPO	PUF	Mixed with cathode‐active material (LiFePO_4_)	[35]	≈160^b)^
Macrocapsule	Pentadecane	PAP	Embedded in a pouch cell	[36]	–
Integrated capsule	TPP	PVDF‐HFP	The core of separator microfibers	[37]	≈200^b)^
	PW	PAN	The core of separator nanofibers	[39]	–
	PI–TPP	Cu	The core of CCs	[38]	≈244^c)^
	PI–TPP	Al	The core of CCs	[38]	≈244^c)^

^a)^ Boiling points of DMTP@PMMA microcapsules

^b)^ Onset temperature of weight loss

^c)^ Boiling point of TPP.

In summary, TRR capsule‐based batteries have bionic features, because TRR capsules are similar in structure and function to core–shell forms in nature. They also have smart functions, because TRR capsules can be stimulated by specific temperatures or other factors to release TRRs.

## 3 Mechanism of TRRs in Suppressing TR

**Figure 2:**
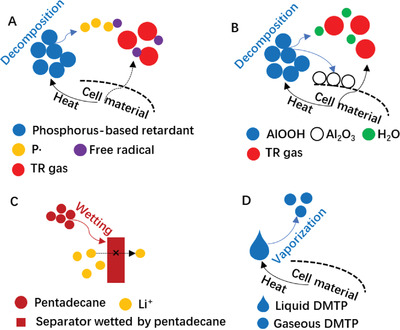
Mechanism of TRRs in batteries. A) Phosphorus‐based fire retardants. B) Boehmite. C) Electrolyte poisons. D) Other TRRs.

According to the types of TRRs, the mechanism of suppressing battery TR is summarized, as shown in **Figure** **2**.

### 3.1 Phosphorus‐Based Fire Retardants

Many fire retardants such as TPP, DOPO, and TCP contain phosphorus compounds, which are efficient radical scavengers. The combustion process is exothermic, powered by free‐radical reactions, and the existence of radical stabilizers impedes combustion, as shown in Figure 2A. Flame retardancy mechanisms were described in detail, as shown in Equations (1)–(7).^[^ ^40^ ^]^ Those containing phosphorus can act in the gas phase. In this case hydrogen and hydroxy radicals are replaced by less effective radicals or are rendered harmless by radical recombination in the gas phase. Some key reactions, of the hundreds possible, are proposed to be the most important (Equation (1)). Branching and chain reactions of the oxidation of hydrocarbons in the gas phase are slowed down or interrupted, which is called flame inhibition, and reduces the production of heat. It is believed that the PO‐radical plays the major role. This process can prevent or suppress the third stage of the TR process

(1) $$PO^{\cdot }+H^{\cdot }\to \mathrm{HPO}$$

(2) $$PO^{\cdot }+OH^{\cdot }\to \mathrm{HP}O_{2}$$

(3) $$\mathrm{HPO}+H^{\cdot }\to H_{2}+{\mathrm{PO}}^{\cdot }$$

(4) $$OH^{\cdot }+H_{2}+PO^{\cdot }\;r\to H_{2}O+\mathrm{HPO}$$

(5) $$\mathrm{HP}{O_{2}}^{\cdot }+H^{\cdot }\to H_{2}O+\mathrm{PO}$$

(6) $$\mathrm{HP}{O_{2}}^{\cdot }+H^{\cdot }\to H_{2}+PO_{2}$$

(7) $$\mathrm{HP}{O_{2}}^{\cdot }+OH^{\cdot }\to H_{2}O+PO_{2}$$

### 3.2 Inorganic Metal Hydroxide Fire Retardants

Inorganic metal hydroxide fire retardants are popular because they can absorb much heat. Boehmite (AIOOH) belongs to inorganic‐based metal hydroxides. Its decomposition is a highly endothermic reaction, forming alumina (Al_2_O_3_) powder and releasing water while absorbing significant heat, as shown in Figure 2B and Equation (8). The released water both lowers the temperature and dilutes combustible gases. The residual Al_2_O_3_ powder forms a protective barrier against further decomposition of the material to reduce the heat‐releasing rate.^[^ ^34^ ^]^ Because the decomposition temperature of boehmite is ≈400 °C, it can be used to suppress the third stage of the TR process

(8) $$2\;\mathrm{AlOOH}\overset{\mathrm{heat}}{\to }Al_{2}O_{3}+H_{2}O$$

### 3.3 Electrolyte Poisons

**Figure 3:**
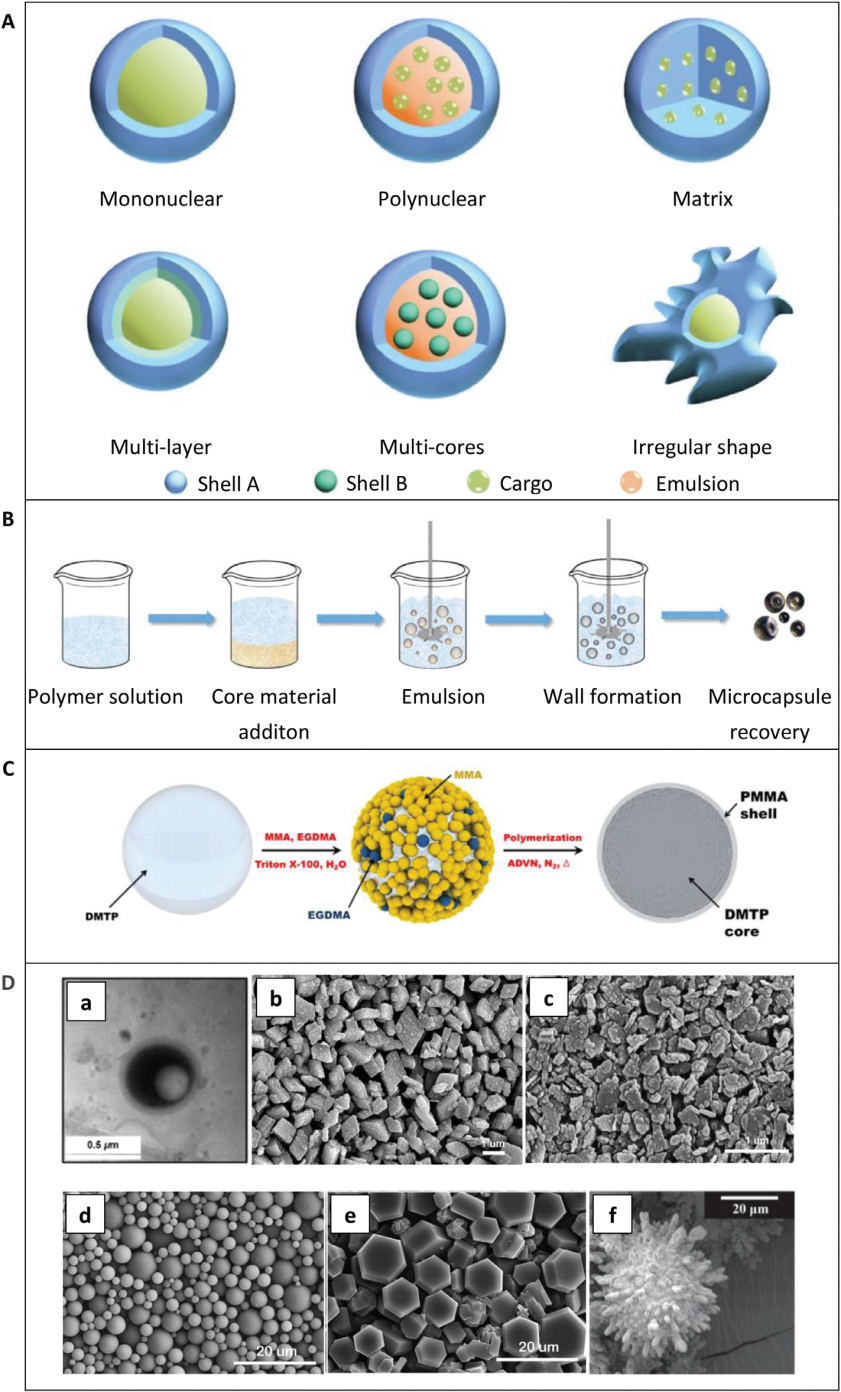
Microencapsulation process via in situ polymerization and images of microcapsules. A) Microcapsules have regular or irregular shapes, and mononuclear or multinuclear structures with one or several layered shells can be obtained. Reproduced with permission.^[^ ^26^ ^]^ Copyright 2019, Springer Nature Switzerland AG. B) Illustration of the microencapsulation process via in situ polymerization. Reproduced with permission.^[^ ^25^ ^]^ Copyright 2018, Elsevier Inc. C) Schematic illustrations for the synthesis route of the DMTP microcapsules. Reproduced with permission.^[^ ^32^ ^]^ Copyright 2015, American Chemical Society. D) SEM images of microcapsules. a) DMTP microcapsules. Reproduced with permission.^[^ ^32^ ^]^ Copyright 2015, American Chemical Society. b) AlOOH‐L microcapsules. c) AlOOH‐S microcapsules. b,c) Reproduced with permission.^[^ ^34^ ^]^ Copyright 2017, Elsevier Inc. d) TPP microcapsules. e) DOPO microcapsules. d,e) Reproduced with permission.^[^ ^35^ ^]^ Copyright 2017, Elsevier Inc. f) TCP microcapsules. Reproduced with permission.^[^ ^33^ ^]^ Copyright 2018, American Chemical Society.

Electrolyte poisons includes pentadecane, THA, BA, DBA, and diols, diamines, and BMI. The mechanism of pentadecane suppressing TR is shown in **Figure** **3**D. Because pentadecane is immiscible with battery electrolytes and is more wettable to the separator than electrolytes are, it will repel the electrolyte and form a physical blocking layer in the separator, suppressing Li^+^ transport. Charge transfer reactions at the electrode–electrolyte interface is suppressed due to the accumulation of reaction products.^[^ ^36^ ^]^ The mechanism of other electrolyte poisons on battery TR is similar: THA is highly wettable to the separator and immiscible with the electrolyte, and therefore, it blocks Li^+^ transport; BA and DBA decrease the ionic conductivity of the electrolyte and increase the charge transfer resistance; diols hinder charge carrying‐ion mobility by raising solution viscosity, while diamines disrupt solvent permittivity by rapidly polymerizing the ethylene carbonate (EC) solvent; and BMI can polymerize to cause rapid solidification of the electrolyte at 110 °C.^[^ ^21^, ^22^, ^23^, ^24^ ^]^ This process can prevent or suppress the first and second stages of the TR process.

### 3.4 Other TRRs

As extinguishing agent, DMTP can be vaporized in a timely manner by the absorption of external heat and finally extinguishes the fire before the cell reaches a serious TR state. The latent heat of DMTP is 102.1 J g^−1^ and the boiling point is ≈88 °C. It has remarkable endothermic properties in an LIB to effectively suppress a drastic temperature rise.^[^ ^32^ ^]^

Phase change materials can absorb heat through physical changes, delaying or avoiding the occurrence and development of battery TR. It was reported PW (solid at room temperature) was used in capsules to suppress TR of LIBs.^[^ ^39^ ^]^ Its latent heat is 240 J g^−1^ and melting point is ≈45 °C.

With further in‐depth and extensive research, more kinds of TRRs are expected to be used in capsules, which will also further expand the mechanisms of inhibiting TR.

## 4 Synthesis of Capsules

### 4.1 Microcapsules

Figure 3 shows microencapsulation process via in situ polymerization and images of microcapsules. Generally, microcapsules are regular spheres and only a few nonspherical microcapsules have been reported.^[^ ^41^ ^]^ Multiple forms of microcapsules can be obtained according to shapes, layers of wall materials, or number and position of cores, as shown in Figure 3A.^[^ ^26^ ^]^ Microcapsule preparation includes polymerization, phase separation, and physical–mechanical methods. Polymerization reaction methods include interfacial polymerization, in situ polymerization, suspension crosslinking, and so on.^[^ ^25^ ^]^ At present, in situ polymerization is generally used to prepare microcapsules for battery TRRs.^[^ ^32^, ^33^, ^34^, ^35^ ^]^

In the process of preparing microcapsules by in situ polymerization, the capsule shell is formed by the polymerization of monomers, which generally involves the initial dispersion or emulsion of the core material, followed by capsule‐wall deposition, and ultimately recovery of the microcapsules, as shown in Figure 3B.^[^ ^25^ ^]^ Capsule‐wall deposition includes three important processes as summarized:^[^ ^42^ ^]^ 1) dissolution of the monomers in the continuous phase; 2) polymer formation; and 3) precipitation of the polymer and deposition at the interface. The reactive monomers and initiators are added to the dispersed or continuous phase, that is, the monomers and initiators are located inside or outside the liquid core material (the dispersed phase). The monomers are soluble in a single phase, while their polymer is insoluble in the entire system. Therefore, the polymerization reaction occurs in the dispersed phase, that is, on the surface of the core material. Monomer polymerization produces a relatively low molecular weight prepolymer. As the size of the prepolymer gradually increases by continued polymerization, it is deposited on the surface of the core material. Due to the continuous progress of crosslinking and polymerization, a solid capsule shell forms. The resulting polymer film, the capsule shell, covers the entire surface of the core.^[^ ^39^ ^]^ For example, DMTP droplets were encapsulated with a rigid PMMA shell via an oil‐in‐water emulsion‐based polymerization reaction using methyl methacrylate (MMA) monomer, ethylene glycol dimethacrylate (EGDMA) as a crosslinking agent, and 2,2‐azobis(2,4‐dimethylvaleronitrile) (ADVN) as a polymerization initiator, as shown in Figure 3C.^[^ ^34^ ^]^ The morphologies of the microencapsulated TR inhibitors listed Table 1 are shown in Figure 3D. The figure shows the diversity of microcapsule shapes, including spherical (Figure 3D‐a,d,f), rhomboid (Figure 3D‐b), fractal plate (Figure 3D‐c), or hexagonal plate (Figure 3D‐e). The surface roughness of the microcapsules also varies due to the different preparation processes. The core–shell structure of DMTP microcapsules can be clearly seen in Figure 3D‐a.

**Figure 4:**
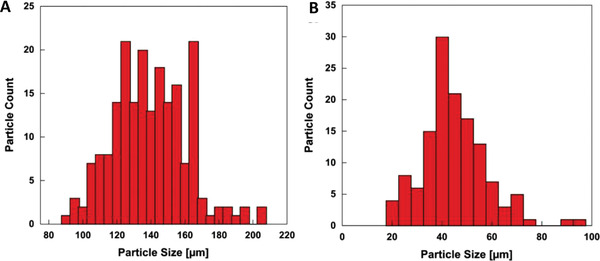
Size distribution of TCP microcapsules. A) TCP microcapsules prepared at 1500 rpm. B) TCP microcapsules prepared at 3000 rpm. Reproduced with permission.^[^ ^33^ ^]^ Copyright 2018, American Chemical Society.

Microcapsule size is closely related to the TRR type and preparation process, as shown in **Figure** **4**. The TPP microcapsules have an average size of 2–8 µm as shown in Figure 3D‐d, and the DOPO microcapsules have an average size of 4–12 µm as shown in Figure 3D‐e. After completion of the reaction, TCP microcapsules were centrifuged in water to remove excess surfactant and filter dried in air.^[^ ^33^ ^]^ Microcapsules produced using this procedure have average diameters of 137 and 43 µm when prepared at 1500 and 3000 rpm, respectively.

### 4.2 External Capsules

The preparation of external capsules mainly adopts methods such as thermal sealing. To prevent air from entering the capsule, capsules are sealed in a glove box.^[^ ^36^ ^]^ No literature has been found to describe the preparation process of TRR external capsules in detail.

### 4.3 Integrated Capsules

**Figure 5:**
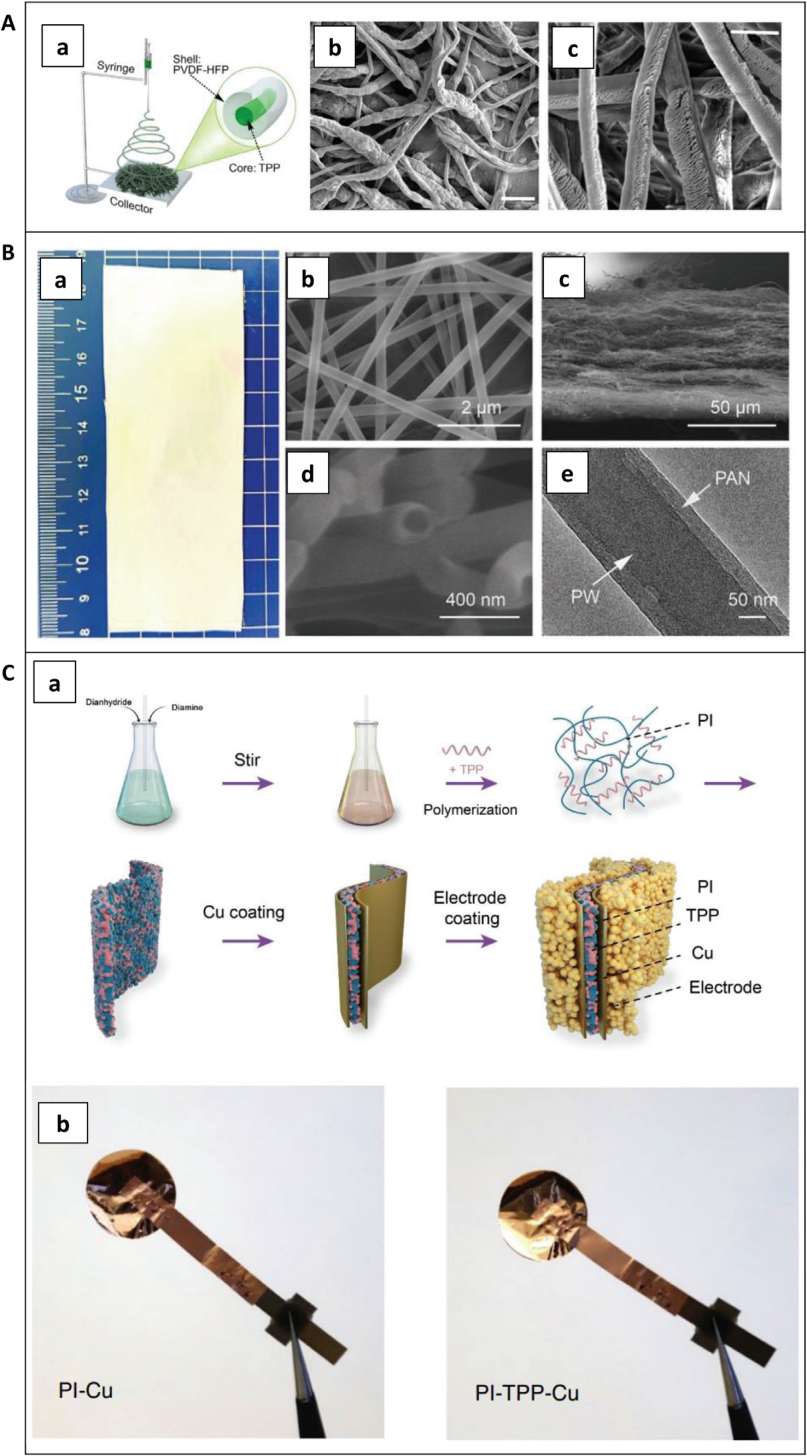
Synthesis process and images of integrated capsules. A) Synthesis schematic and microstructures of the TPP@PVDF‐HFP separator. a) Schematic illustration for microfiber fabrication by electrospinning. b) SEM image of TPP@PVDF‐HFP microfibers (scale bar: 5 µm). c) SEM image of TPP@PVDF‐HFP microfibers after etching (scale bar: 5 µm). a‐c) Reproduced with permission.^[^ ^37^ ^]^ Copyright 2017, American Association for the Advancement of Science. B) Morphology and microstructure of PW@PAN nanofibers. a) Photograph. b) SEM image of the surface. c) SEM image of the cross‐section. d) SEM image of a cut surface. e) TEM image. a‐e) Reproduced with permission.^[^ ^39^ ^]^ Copyright 2021, Wiley‐VCH. C) Synthesis process and morphology of PI–TPP–Cu CC‐based electrode capsules. a) The overall synthesis schematic. b) Digital camera photos showing the welding of PI–Cu and PI–TPP–Cu CCs onto copper foil. a,b) Reproduced with permission.^[^ ^38^ ^]^ Copyright 2020, Springer Nature.

An integrated capsule can be prepared by filling the TRR into a cell component. This means that the shell of the integrated capsule is an empty component of a cell, and the component is then filled with TRRs (i.e., the core of the capsule). Therefore, the synthesis of integrated capsules is closely related to that of the corresponding cell component. Two types of integrated capsules have been reported in literature, separator type and CC type. **Figure** **5** shows the synthesis process and images of integrated capsules.

For separator type TPP integrated capsules, TPP@PVDF‐HFP fiber was fabricated by electrospinning,^[^ ^37^ ^]^ and is shown in Figure 5A. During synthesis, TPP and PVDF‐HFP were dissolved in a solvent mixture of dimethylacetamide and acetone. Then, the solution was placed into a syringe with a stainless‐steel needle. A voltage of 13 kV was applied to the solution using a commercial high voltage source to start the electrospinning process, as shown in Figure 5A‐a. Scanning electron microscope (SEM) images of the integrated capsules based on separators are shown in Figure 5A‐b,c. Nanoflakes stacked inside the fibers can be clearly observed from Figure 5A‐c.

Separator‐based integrated capsules prepared with phase‐changing material as the core are shown in Figure 5B. As shown in Figure 5B‐a, PW@PAN nanofibers were prepared by coaxial electrospinning.^[^ ^39^ ^]^ The precursor solution for the sheath layer was obtained by dissolving PAN in dimethyl fumarate. The core precursor solution was prepared by adding melted paraffin to kerosene. Then, both the sheath and core solutions were separately loaded into plastic syringes, which were connected to a dual‐needle with outer and inner stainless‐steel needles. The SEM images show that the PW@PAN membranes are formed by interconnected nanofibers with an average diameter of ≈280 nm with a bead‐free structure, as shown in Figure 5B‐b. Numerous continuous and interlinked nanopores can be observed on the surface and cross‐section of the PW@PAN nanofibers, as shown in Figure 5B‐b,c. The PW@PAN nanofibers exhibited an obvious core–sheath structure, indicating that PW was successfully encapsulated in the PAN nanofiber sheath with hollow interiors, as shown in Figure 5B‐d,e.

Integrated capsules based on CCs with built‐in TRRs are mainly obtained by polymerization and coating,^[^ ^38^ ^]^ as shown in Figure 5C‐a. During the synthesis process, polyamic acid (PAA) was mixed with TPP. PAA–TPP films were obtained by polymerization. As‐obtained free‐standing PAA–TPP films were imidized in a box furnace under air exposure to obtain PI–TPP films. Cu/Al layers were then deposited on the PI–TPP films by pulsed DC magnetron sputtering using a Cu/Al target under a protective argon atmosphere. Integrated capsule films based on CCs are shown in Figure 5C‐b.

## 5 Release Property of TRRs

The release property of TRRs, i.e., the core material of the capsules, is of great importance in simultaneously improving safety and electrochemical performance of LIBs. Releasing TRRs at too low temperatures will worsen the battery electrochemical performance, and at too high temperatures, it will not be able to effectively suppress the TR reactions. The ability of the microcapsules to release their content in response to exposure to a critical (trigger) temperature can be assessed indirectly by differential scanning calorimetry (DSC) and thermogravimetric analysis (TGA), and examined directly by nuclear magnetic resonance (NMR) and ultraviolet–visible absorbance spectrum.

**Figure 6:**
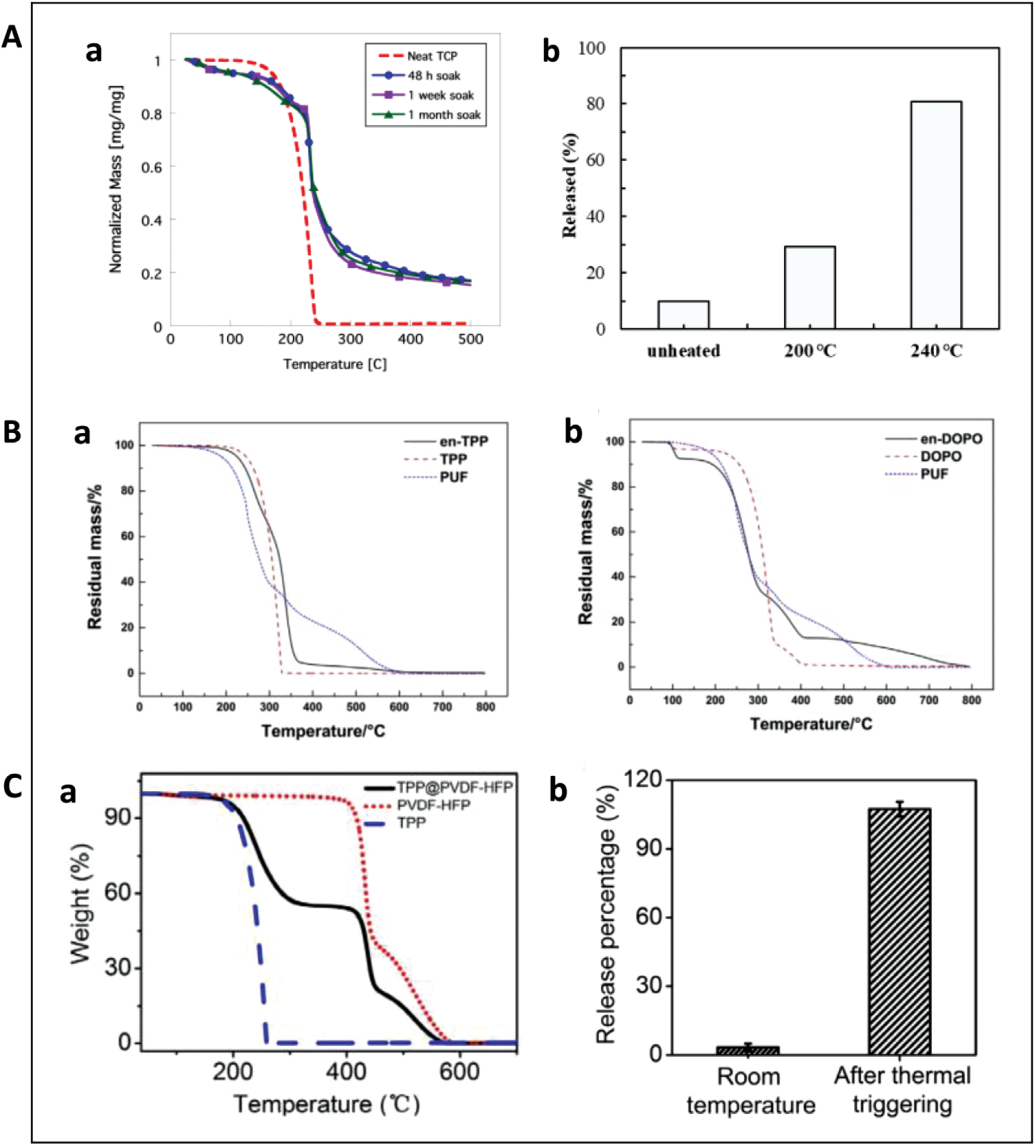
Release property of TRRs. A) Release property of TCP microcapsules. a) Thermogravimetric analysis for TCP microcapsules. Neat capsules were prepared at 3000 rpm and were soaked in the LiClO_4_ EC/DMC electrolyte for 48 h, 1 week, and 1 month before TGA scanning at 10 °C min^−1^. Reproduced with permission.^[^ ^33^ ^]^ Copyright 2018, American Chemical Society. b) NMR results for TCP release from microcapsules prepared at 1500 rpm (≈137 µm diameter) upon exposure to high temperature. The graph is based on data in ref. [33]. B) Thermogravimetric weight loss as a function of temperature for TPP microcapsules, TPP, PUF, DOPO microcapsules, and DOPO. a) TPP microcapsules, TPP, and PUF. b) DOPO microcapsules, DOPO, and PUF. C) Release property of TPP@PVDF‐HFP separator, PVDF‐HFP, and TPP. a,b) Reproduced with permission.^[^ ^35^ ^]^ Copyright 2017, Elsevier Inc. a) Thermogravimetric analysis for TPP@PVDF‐HFP separator, PVDF‐HFP, and TPP. b) The percentage of TPP being released into the electrolyte before and after thermal triggering at 160 °C. The calculated percentage after thermal triggering is slightly above 100%, possibly because of the evaporation of solvent during heating. a,b) Reproduced with permission.^[^ ^37^ ^]^ Copyright 2017, American Association for the Advancement of Science.

For DMTP@PMMA microcapsules, the glass transition temperature and boiling point is 95 and 127 °C shown by a DSC test result.^[^ ^27^ ^]^ The boiling point is higher than those of DMTP (88 °C) and PMMA (122 °C). For TCP@PUF microcapsules, the mass loss prior to 100 °C is attributed to residual moisture trapped in the rough surface morphology, and the total mass loss from 130 to 400 °C was taken as a measure of TCP content, as shown in **Figure** **6**A‐a.^[^ ^28^ ^]^ For the unheated sample TCP@PUF microcapsules, only ≈9.8% core release was measured from the NMR results as shown in Figure 6A‐b.^[^ ^33^ ^]^ Upon exposure to 200 °C, nearly 30% core release occurs, and at 236 °C nearly complete (80.9%) core (TCP) release was measured. For TPP@PUF microcapsules, the first weight loss is at ≈170 °C, between those of TPP (120 °C) and PUF (200 °C), as shown in Figure 6B‐a.^[^ ^35^ ^]^ For DOPO@PUF microcapsules, the onset temperature at ≈160 °C is much lower than those of DOPO (210 °C) and PUF (200 °C), as shown in Figure 6B‐b.^[^ ^35^ ^]^ From the above results, it can be concluded that first weight loss temperature has no obvious regularity between the microcapsule and the microcapsule shell/core. However, microcapsules in general exhibit the combined decomposition behavior of the microcapsule shell and core, as shown in Figure 6B‐a,C‐a,D.

For TPP@PVDF‐HFP integrated capsules, the substantial weight loss started at ≈200 °C, higher than that of TPP (≈150 °C), as shown in Figure 6C‐a.^[^ ^37^ ^]^ When the TPP@PVDF‐HFP fiber was soaked in the EC/diethyl carbonate (DEC) electrolyte and stored at room temperature (≈25 °C), the release of TPP into the electrolyte was only ≈4%. However, upon heating up to 160 °C, above the melting points of TPP (≈50 °C) and PVDF‐HFP (≈150 °C), all of the encapsulated TPP ≈100%) was abruptly released into the electrolyte monitored quantitatively by ultraviolet‐visible absorbance spectrum, as shown in Figure 6C‐b.^[^ ^37^ ^]^ In another study, TPP begins to decompose at ≈250 °C and is completely removed from the sample by 350 °C, while the decomposition of PI mainly occurs between ≈550 and ≈650 °C.^[^ ^38^ ^]^ The TGA curve of PI–TPP is the coupling of PI and TPP. For PI–TPP@Cu/Al integrated capsules, the mismatched temperature coefficients of PI and Cu/Al will result in breakage of the original planar structure and cause cracks during TR, which will release TPP or TPP radicals. At temperatures above 244 °C (the boiling point of TPP), the flame retardant will be gasified and quickly released.^[^ ^38^ ^]^

In addition, for external capsules, the capsule shell is ruptured by a nail reported by Shi et al., thus releasing TRRs mechanically.^[^ ^36^ ^]^

In summary, the release temperature can be approximately characterized by boiling point, glass transition temperature, initial weight loss temperature, etc. The release temperatures of TRRs for different types of capsules are shown in Table 1, ranging between 127 and 244 °C. This indicates that the release temperature of TRRs is lower than the normal operating temperature of the battery. However, it can be seen from Figure 6A‐b,C‐b that a small amount of TRR is still released into the electrolyte at room temperature, but the content is too small to deteriorate battery performance.^[^ ^33^, ^37^ ^]^ If more TRRs are mistakenly released from the capsules, the electrochemical performance of the battery will be generally deteriorated. Therefore, the electrochemical performance can be used as an important parameter to indirectly judge the wrong release of TRR. In addition, sensing the concentration of core materials may be a way to directly detect their mistaking release. More importantly, the design and verification of the capsule should be adequately done so as not to deteriorate the electrochemical performance of the battery during normal operation. Furthermore, the disintegration of the solid‐electrolyte interphase (SEI) will occur, some combustible gases will be liberated due to electrolyte decomposition, the separator will deform or melt and the cathode will decompose from ≈80 °C to above 200 °C.^[^ ^6^, ^7^, ^8^ ^]^ TRRs are released in this temperature range as shown in Table 1, therefore capsule‐based batteries can effectively suppress combustion during TR.

It should be noted that the responding time of the TRR capsules is also an important parameter that needs to be considered. This is because during TR the cell temperature rise rate is very high (up to hundreds of degrees per second or higher), and there exists a heat transfer process from the cell materials to the microcapsules. The responding time of the TRR capsules can be indirectly assessed by a fire extinguishing experiment. For example, when a standard electrolyte was ignited, it continuously burned until the combustible electrolyte was fully exhausted. On the contrary, the electrolyte containing DMTP@PMMA microcapsules showed a markedly different behavior; the flames rapidly diminished within 10 s and the fire was completely extinguished within 20–30 s.^[^ ^32^ ^]^ However, this has not been studied in depth in open literature.

In short, release temperature, response time, etc., are all key parameters of capsule release performance, which play a vital role in battery safety and electrochemical performance. More advanced materials and preparation methods need to be further developed to match the release performance of capsules and the safety and electrochemical performance of batteries.

## 6 Safety and Electrochemical Performance

Lightweight batteries have always been an urgent need in the electric vehicle market to achieve cost savings. The specific energy of the power battery, which is inversely related to its weight, is far from ideal. Therefore, the design of high‐specific energy batteries is a hot spot for continuous future research. However, high‐specific energy means high activity of battery materials, and high activity means low safety. High‐safety batteries tend to have lower electrochemical performance, which in turn decreases the specific energy. Batteries based on TRR capsules make up for this shortcoming. To show the advantages of TRR capsule technology in solving this problem, this review summarizes the performance of TRR capsule‐based batteries from the two perspectives of cycle capacity retention rate or specific energy change, and safety.

### 6.1 LIBs with Microcapsules

**Figure 7:**
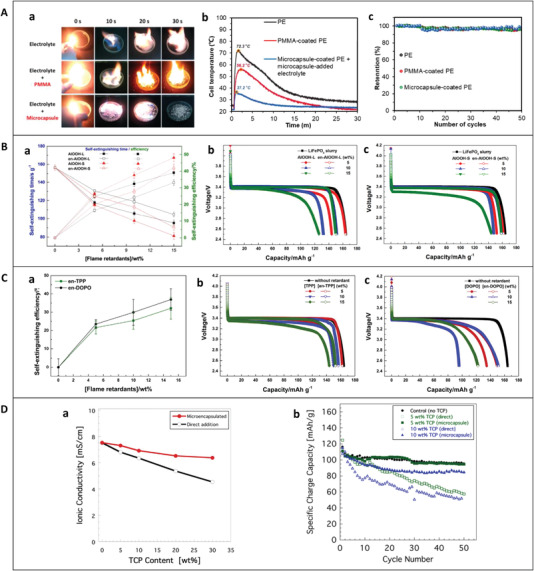
Safety and electrochemical performance of cells with microcapsules. A) DMTP microcapsules. a) Fire retardancy test results for the DMTP microcapsules. b) Temperature profiles during the nail penetration test of the full cell (graphite/LiNi_0.5_Co_0.2_Mn_0.3_O_2_(NCM_523_)) with DMTP microcapsules. The cells were fully charged to 4.35 V before testing. c) Capacity retention test up to 50 cycles of the full cell (graphite/NCM523) with DMTP microcapsules. a‐c) Reproduced with permission.^[^ ^32^ ^]^ Copyright 2015, American Chemical Society. B) AlOOH‐L microcapsules and AlOOH‐S microcapsules. a) Self‐extinguishing time (left axis) and efficiency (right axis) of the cathode/electrolyte mixture as a function of the content of AlOOH‐L, AlOOH‐S, AlOOH‐L microcapsules, and AlOOH‐S microcapsules. b) Discharge curves at 0.1C for cells constructed with LiFePO_4_ cathodes without and with the addition of AlOOH‐L and AlOOH‐L microcapsules at contents of 5, 10, and 15 wt%. c) Discharge curves at 0.1C for cells constructed with LiFePO_4_ cathodes without and with the addition of AlOOH‐S and AlOOH‐S microcapsules, at contents of 5, 10, and 15 wt%. a‐c) Reproduced with permission.^[^ ^34^ ^]^ Copyright 2017, Elsevier Inc. C) TPP microcapsules and DOPO microcapsules. a) Self‐extinguishing efficiency of the cathode/electrolyte mixtures as functions of microcapsule content. b) Discharge curves at 0.1C for cells constructed with LiFePO_4_ cathodes without and with the addition of TPP microcapsules at contents of 5, 10, and 15 wt%. c) Discharge curves at 0.1C for cells constructed with LiFePO_4_ cathodes without and with the addition of DOPO microcapsules, at contents of 5, 10, and 15 wt%. a‐c) Reproduced with permission.^[^ ^35^ ^]^ Copyright 2017, Elsevier Inc. D) TCP microcapsules. a) Effect of flame retardant (TCP) on the Li‐ion pouch cell cycling performance at 1 °C, with LiClO_4_ EC/DMC electrolyte. Microcapsule fabrication at 3000 rpm (≈43 µm diameter). b) Ionic conductivity and self‐extinguishing time as a function of TCP content. a,b) Reproduced with permission.^[^ ^33^ ^]^ Copyright 2018, American Chemical Society.

Due to the extensive development of microencapsulation technology, there are more applications of microcapsules than other types of capsules. Microcapsules can be blended with the electrode material, mixed with the electrolyte, or coated on the separators of a cell. **Figure** **7** shows the safety and electrochemical performance of TRR microcapsule‐based batteries.

In 2015, Yim et al. used DMTP as the core and temperature‐responsive polymeric layers as the shell to prepare DMTP@PMMA microcapsules.^[^ ^32^ ^]^ To verify the influence of DMTP@PMMA microcapsules on electrolyte flammability, they carried out flame retardant experiments on standard electrolytes, electrolytes with PMMA, and electrolytes with DMTP@PMMA microcapsules and found that the self‐extinguishing time (SET) values of these were 40.0, 36.7, and 10.0 s g^−1^, as shown in Figure 7A‐a. The DMTP@PMMA microcapsules significantly reduced electrolyte flammability. They coated the DMTP@PMMA microcapsules on a polyethylene (PE) separator (mass loading of microcapsules = 0.636 g g^−1^ of PE) and dispersed them (10 wt%) in the electrolyte to prepare a graphite/LiNi_0.5_Co_0.2_Mn_0.3_O_2_ (NCM523), 500 mAh pouch cell. For comparison, cells with a pristine PE separator and a PMMA‐coated PE separator were also prepared with the standard electrolyte and tested. In the nail penetration test, the highest temperatures of the cells using a pristine PE separator, a PMMA‐coated PE separator, and a DMTP@PMMA microcapsule‐coated PE separator were 72.3 °C, 56.2 °C, and 37.2 °C, respectively, as shown in Figure 7A‐b. Compared with the PE separator and the PMMA‐coated PE separator, the maximum temperature of the cell with microcapsules has dropped by 48.5% and 33.8%. After 50 cycles, the capacity retention rates of the three types of cells were all above 95%, with no obvious differences between them. The DMTP@PMMA microcapsules did not adversely affect the electrochemical performance of the cell, as shown in Figure 6A‐c. In addition, assuming that the electrolyte accounts for 10 wt% and the separator accounts for 3 wt% of the battery, it can be calculated that the DMTP@PMMA microcapsules account for 2.9% of the battery weight.

In 2017, Huang et al. used the flame retardants AlOOH, TPP, and DOPO as the cores and PUF as the shell to form microcapsules, and doped with LiFePO_4_, electrolyte, etc.^[^ ^34^, ^35^ ^]^ They found that whether AlOOH is microencapsulated or not, as its content increases, the SET of the cathode/electrolyte mixture is significantly reduced, and the self‐extinguishing efficiency is significantly increased, as shown in Figure 7B‐a. It can be seen from the charge–discharge curve that, at the same AlOOH content, the plateau voltage of the battery with TRR microcapsules has a lower drop and a higher specific capacity, as shown in Figures 6B‐c and 7B‐b. This is because of the better contact among cathode materials due to the encapsulated flame retardant. They found that the electrode/electrolyte had better flame‐retardant performance after adding microcapsules, and the electrochemical performance of the electrode and cell was not significantly reduced when the additives used were 5–15 wt% of LiFePO_4_ power. In terms of SET and charge‐discharge curves, similar results were obtained for TPP and DOPO, as shown in Figure 7C‐a–c. In addition, assuming that the positive electrode material accounts for ≈20% of the battery weight, the amount of TRR microcapsules accounts for ≈1% to 3% of the cell weight.

In 2018, Baginska et al. used flame retardant TCP as the core and PUF as the shell to prepare TCP@PUF microcapsules.^[^ ^33^ ^]^ Li(Ni_1/3_Co_1/3_ Mn_1/3_)O_2_ (Li333) is used as the cathode, mesocarbon microbeads are used as the anode, and LiClO_4_ EC/dimethyl carbonate (DMC) is used as the electrolyte to prepare a pouch cell. When the same amount of TCP is used, the ionic conductivity of the LiClO_4_ EC/DMC electrolyte is significantly higher after microencapsulation, as shown in Figure 7D‐a. The cell specific capacity decreased by ≈42% after 50 cycles after adding 5% TCP directly to the electrolyte, compared to electrolyte without TCP. In contrast, with the same quantity of TCP@PUF microcapsules, the cell cycle specific capacity hardly changed. The cycle specific capacity of the cells with 10% TCP and 10% TCP@PUF microcapsules decreased by ≈47% and 11%, respectively, as shown in Figure 7D‐b. These results indicate that the capacity retention rate of the cell with TCP@PUF microcapsules is significantly higher than that with unencapsulated TCP. They also found that sequestration of the flame retardant within the polymeric microcapsule, isolated from the electrolyte, is an effective approach to maintaining ionic conductivity while simultaneously reducing electrolyte flammability. In addition, assuming that the weight of the electrolyte accounts for 10% of the weight of the battery, it can be calculated that the amount of TCP@PUF microcapsules accounts for 0.5% of the weight of the cell.

In short, because the microcapsules can be completely dispersed inside the cell, a smaller amount of TRRs can significantly improve cell safety without affecting electrochemical performance. However, due to different test conditions and safety evaluation methods, it is impossible to compare which TRR is more suitable for LIBs.

### 6.2 LIBs with External Capsules

**Figure 8:**
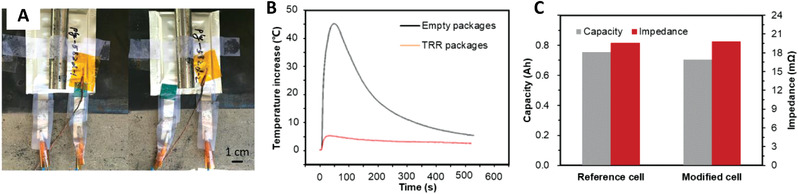
Safety and electrochemical performance of cells with external capsules. A) Pouch cells embedded with empty packages and TRR external capsules. B) Temperature profiles of pouch cells in impact test. C) Capacity and impedance of pouch cells embedded with empty external capsules and TRR external capsules. A‐C) Reproduced with permission.^[^ ^36^ ^]^ Copyright 2017, AIP Publishing LLC.

**Figure** **8** shows the safety and electrochemical performance of TRR external capsule‐based batteries. LIBs with external capsules means that TRR is packaged, and then the package, i.e., the external capsule, is placed inside the battery, as shown in Figure 8A. There are few studies on batteries related to external capsules. In 2017, Shi et al. filled pentadecane into a package with PAP as the shell material to produce a cylindrical pentadecane@PAP external capsule.^[^ ^36^ ^]^ The diameter of the external capsule is 6 mm and the length is 35 mm. Four pentadecane@PAP external capsules are embedded in a NCM523 soft‐package cell. Compared with the reference cell using an empty external capsule without pentadecane, the maximum increase in cell temperature decreased by 90% (only increased by 5 °C) in the nail penetration test, as shown in Figure 8B. The capacity of the modified cell with external capsules has lost 6.4% and the impedance has increased by 1.0%, as shown in Figure 8C. In addition, the whole external capsule content is 5 wt% of the cell.

Because the TRR is encapsulated in a large capsule, it cannot be fully mixed with the battery material in time after being released from the external capsule, so the required amount of TRR is higher (5%), which significantly impacts the specific energy of the battery. However, an obvious advantage of the external capsule is that it can be processed separately without the need to change any cell structures. In addition, external capsules are relatively simple to make and can encapsulate more types of TRRs than microcapsules. Therefore, combined with high‐specific energy technology, TRR external capsule technology has promising application prospects.

### 6.3 LIBs with Integrated Capsules

**Figure 9:**
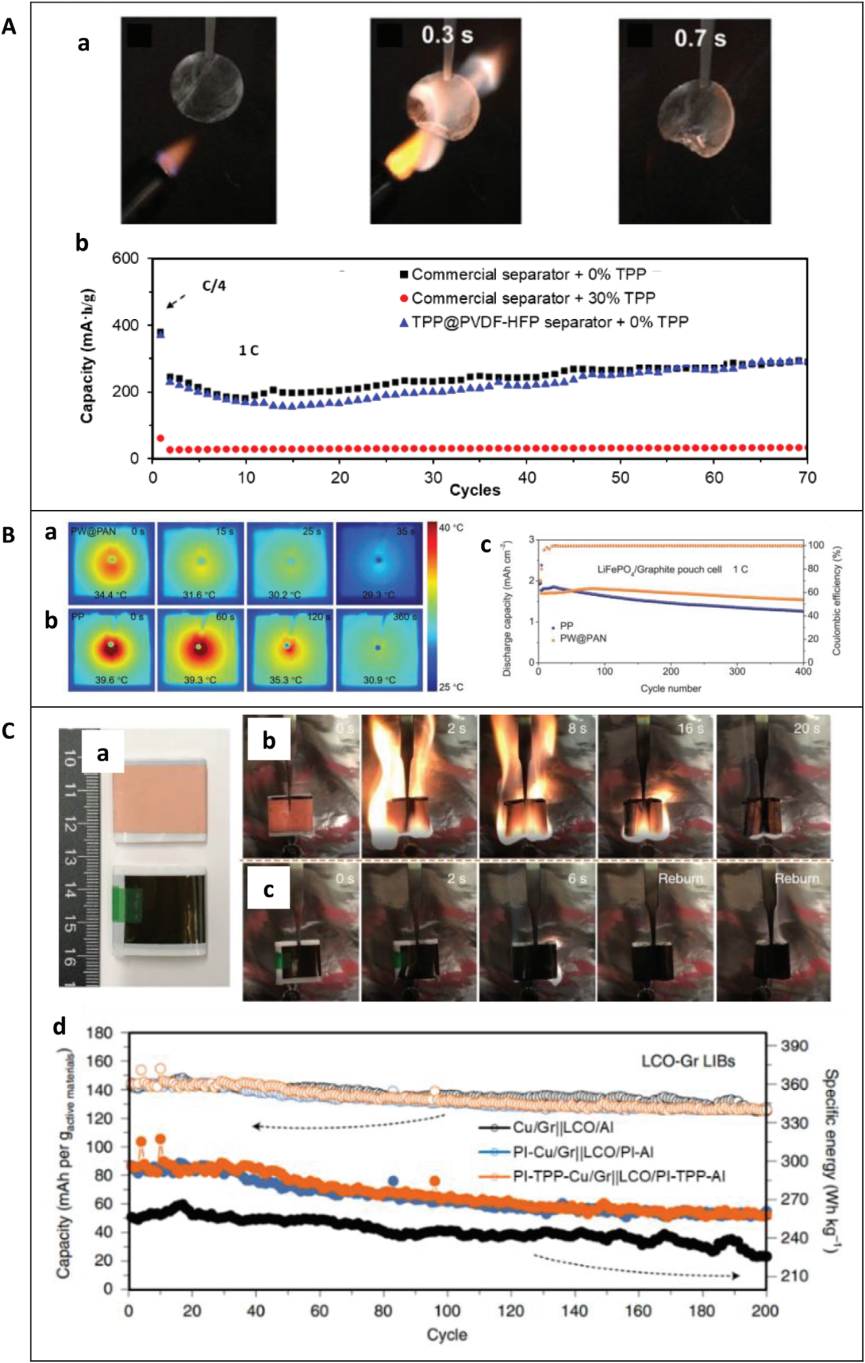
Safety and electrochemical performance of cells with integrated capsules. A) TPP integrated capsules based on separators. a) Digital photographs showing the flammability of the TPP@PVDF‐HFP separator wetted by the electrolyte. The respective times, counted from when the electrolyte started to burn, are indicated in each picture. The diameter of the separator is 1.6 cm. b) Delithiation capacities of the graphite anode during galvanostatic cycling between 0.01 and 1.5 V. The rate was 0.25 capacity (C) for the first cycle and 1C for subsequent cycles. a,b) Reproduced with permission.^[^ ^37^ ^]^ Copyright 2017, American Association for the Advancement of Science. B) PW integrated capsules based on separators. a) Thermal imaging of the LiFePO_4_/separator/Li pouch cell with a PW@PAN separator before and after the nail penetration test. b) Thermal imaging of the LiFePO_4_/separator/Li pouch cell with a Celgard polypropylene (PP) separator before and after the nail penetration test. c) Cycling performance of LiFePO_4_/graphite pouch‐type full cell. a‐c) Reproduced with permission.^[^ ^39^ ^]^ Copyright 2021, Wiley‐VCH. C) TPP integrated capsules based on current collectors. a) Photos of the assembled Cu/Gr||LCO/Al (top) and PI–TPP–Cu/Gr||LCO/PI–TPP–Al (bottom) pouch full cells. b) Flame retardancy test results for the Cu/Gr||LCO/Al pouch full cells. c) Flame retardancy test results for the PI–TPP–Cu/Gr||LCO/PI–TPP–Al pouch full cells. d) Galvanostatic cycling of Cu/Gr||LCO/Al (black), PI–Cu/Gr||LCO/PI–Al (blue), and PI–TPP–Cu/Gr||LCO/PI–TPP–Al (orange) full cells at 0.5 C. The top three curves are associated with the left *y*‐axis (indicated by the left‐pointing dashed arrow) and the bottom three curves are associated with the right *y*‐axis (indicated by the right‐pointing dashed arrow). The open circles represent the capacity calculated using the mass of the cathode‐active materials and the filled circles represent the capacity calculated using the whole battery mass (without packing). a‐d) Reproduced with permission.^[^ ^38^ ^]^ Copyright 2020, Springer Nature.

LIBs with integrated capsules refer to a cell in which a certain component, such as a current collector or a diaphragm is made hollow and filled with TRRs. **Figure** **9** shows the safety and electrochemical performance of TRR integrated capsule‐based batteries.

In 2017, Liu et al. used the flame retardant TPP, a popular organophosphorus‐based flame retardant, as the core and PVDF‐HFP as the shell to prepared TPP@PVDF‐HFP integrated capsules (i.e., superfine fibers) based on separators.^[^ ^37^ ^]^ The TPP accounts for ≈30 wt% of the electrolyte. After the separator with built‐in TPP was infiltrated with EC/DEC electrolyte, it was ignited by flame. It was found that the electrolyte was completely asphyxiated within 0.4 s, and the corresponding SET value was ≈3 s g^−1^, as shown in Figure 8A‐a. This shows that TPP@PVDF‐HFP integrated capsules based on separators have good flame‐retardant effects on the EC/DEC electrolyte. They found the average cycle specific energy of the carbon anode increased by ≈11 times with integrated capsules based on separators, compared with directly adding TPP to the electrolyte (≈17 mA h g^−1^), as shown in Figure 8A‐b. In 70 cycles, the average specific capacity of the carbon anode is ≈212 mA h g^−1^, which is at the same level (≈233 mA h g^−1^) of the carbon anode using a commercial separator without TPP. Assuming that the electrolyte accounts for 10% of the cell weight, it can be estimated that TRR accounts for 0.3% of the cell weight.

In addition to TRR, the inside of the separator can also be filled with phase‐changing materials. In 2021, Liu et al. prepared hollow polyacrylonitrile nanofibers with phase‐changing PW as the shell and PAN as the wall, and further prepared PW@PAN integrated capsules based on separators.^[^ ^39^ ^]^ During the nail test, compared with the conventional separator, it was found that after adding the PW@PAN integrated capsules based on separators the highest temperature of the cell was lower, and the time required to return to room temperature was shorter, as shown in Figure 9B‐a,b. It can be seen from the cycle curves of LiFePO_4_ pouch batteries that adding PW@PAN integrated capsules based on separators yields a higher cycle retention rate than conventional separators, with similar Coulombic efficiency, as shown in Figure 8B‐c.

In 2020, Ye et al. further prepared a PI‐based CC with built‐in TPP, i.e., TPP–PI@Al/Cu integrated capsules based on CCs.^[^ ^38^ ^]^ TPP accounts for 25 wt% of the total TPP–PI. PI was selected as the CC supporting film due to its low density, superior mechanical properties, good resistance to solvents, excellent thermal stability (>400 °C) and remarkable flame resistance. After being ignited, cells using TPP–PI@Al/Cu integrated capsules based on CCs can be extinguished in a shorter time than batteries using conventional CCs, and the flame during combustion is weaker, as shown in Figure 9C‐a–c. Figure 9C‐d shows the electrochemical performance of the cell using different types of CCs, including traditional CCs, PI‐based CCs and PI–TPP‐based CCs. After 200 cycles, the capacity retention rate of cells with Cu/Gr||LiCoO_2_ (LCO)/Al, PI–Cu/Gr||LCO/PI‐Al, or PI–TPP–Cu/Gr||LCO/PI–TPP‐Al are ≈88.1%, ≈88.0%, and ≈87.2%. This shows that there is no significant change in the cycle capacity retention rate with TPP–PI@Al/Cu integrated capsules based on CCs. In addition, due to the low density of PI, the specific energy of the cell after using PI is significantly higher than that of traditional batteries (PI‐based CCs can increase the specific energy of traditional batteries by ≈16–26%).

The research reviewed here shows that integrated capsule batteries can obtain higher safety by using a smaller amount of TRRs, and it hardly affects the electrochemical performance of the cell. However, there is no unified quantitative evaluation index for safety and electrochemical performance. In this review, we propose the safety variation coefficient (SVC) to quantitatively evaluate cell safety. The SVC of the target cell refers to the percent change in its safety indicator compared to that of the reference cell, as shown in Equation (9). Safety indicators include: self‐extinguishing time; SET value; maximum temperature rise, etc. The lower the safety indicator values of the target cell resulting in larger SVC values, the better the cell safety performance. Therefore, SVC value is positively correlated with cell safety.

In this review, it is proposed to use the electrochemical performance variation coefficient (EVC) to quantitatively evaluate the electrochemical performance of the cell, as in Equation (10). Electrochemical performance mainly refers to the cell specific capacity. The larger the specific capacity of the target cell, resulting in a larger EVC value, the better the electrochemical performance of the cell. Therefore, EVC value is positively correlated with cell electrochemical performance

(9) $$\mathrm{SVC}=\frac{i_{0}-i}{i_{0}}\times 100\%$$

(10) $$\mathrm{EVC}=\frac{i-i_{0}}{i_{0}}\times 100\%$$

where *i*_0_ refers to the safety or the electrochemical performance indicator of the reference cell; and *i* refers to the safety or the electrochemical performance indicator of the target cell.

**Figure 10:**
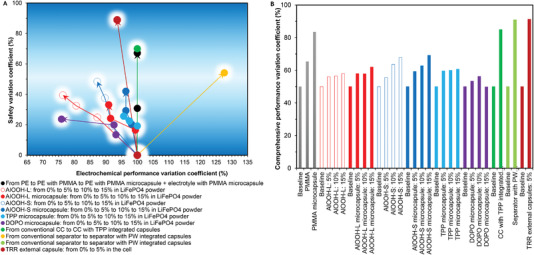
Performance of cells. A) Safety and electrochemical performance of cells. B) Comprehensive performance of cells.

**Figure** **10** shows the performance of batteries with different types of TRR capsules. It can be seen from Figure 10A that when microcapsules are used, the trade‐off between battery safety and electrochemical performance can be significantly improved, as shown by AlOOH‐L and AlOOH‐L microcapsules represented by the red hollow and solid curves, and AlOOH‐S and AlOOH‐S microcapsules represented by the blue hollow and solid curves in Figure 9A. The ideal situation is to greatly improve the cell safety while the electrochemical performance does not decrease or even increase, such as the use of PMMA microcapsules, integrated capsules based on separators and CCs. AlOOH‐S microcapsules, TPP microcapsules, and TRR external capsules can significantly improve cell safety. Although there was an adverse effect on electrochemical performance, it was basically controlled at ≈5%. However, it cannot be concluded that the electrochemical performance would not deteriorate significantly when capsules were used in a cell. For example, when the amount of DOPO microcapsules is increased from 10% to 15%, the safety is hardly improved, but the electrochemical performance is reduced by ≈15%.

Further, to comprehensively evaluate battery performance, this review proposes a comprehensive performance coefficient (CPC) of the cell, as shown in Equation (11), where *x* = *y* = 0.5 in the data in the Figure 10B. In practical applications, *x* and *y*, that is, the contribution rates, can be determined according to specific conditions. It can be seen from Figure 10B that the top four with the highest CPC are PW integrated capsules based on separators, TRR microcapsules, TPP integrated capsules based on CC, and PMMA microcapsules

(11) CPC=x×SVC+y×EVC

where *x* and *y* refer to the contribution rates of SVC and EVC, respectively; *x* + *y* = 1.

Therefore, capsule‐based batteries can take into account the safety and electrochemical performance at the same time, which provides guidance for the future design of high‐specific energy batteries, and also lays the foundation for the application of the next generation of advanced battery technologies, such as high‐nickel technology, high‐specific energy technology, large‐capacity technology, etc.

## 7 Applications of Capsules in the Field of Self‐Healing

Inspired by the autonomous recovery of organisms after being wounded, self‐healing materials have attracted widespread attention during the past several decades. Tremendous efforts have been paid to design and synthesis of novel self‐healing materials that meet requirements for various applications.^[^ ^43^ ^]^ There exist intrinsic and extrinsic strategies according to the self‐healing mechanisms. The former refers to covalent bond and noncovalent‐based self‐healing materials, and the latter are based on capsules, hollow fibers and microvascular networks.^[^ ^43^ ^]^ Newly developed polymeric materials with excellent intrinsic self‐healing functions are good candidates to solve efficiently safety problems of lithium‐based batteries, including dendrite growth, direct contact between anode and cathode electrodes caused by the breakdown of polymer electrolytes and so on.^[^ ^44^ ^]^

In 2001, White systematically proposed the classic theory and technology of self‐healing polymer materials based on microcapsules for the first time.^[^ ^45^ ^]^ Since then, microencapsulation has become a central topic in self‐healing technology, including in battery field. In particular, microencapsulation allows the release of the healing agents. It is suitable for repairing physical and mechanical cracks.^[^ ^43^ ^]^ Therefore, capsule‐based self‐healing is a promising solution to prolong cycling life and improve reliability of LIBs, including self‐healing electrical circuits, self‐healing electrodes, self‐healing electrolytes, self‐healing SEI film.^[^ ^29^, ^46^, ^47^, ^48^, ^49^ ^]^ Furthermore, capsule‐based self‐healing materials can improve the safety of LIBs.

SEI decomposition due to overheating or physical penetration is one of main reactions during battery TR.^[^ ^6^, ^7^, ^8^, ^11^ ^]^ The stable SEI film is beneficial to improve the thermal stability of the anode material, further increase the battery TR initiation temperature, and broaden the safe operating temperature range.^[^ ^6^, ^7^, ^8^, ^11^ ^]^ One of the most common electrolyte additives, vinylene carbonate (VC), can be electrochemically reduced or oxidized on both electrodes to form stable SEI layers consisting of polymeric organic compounds and lithium salts.^[^ ^50^ ^]^ The addition of VC to the electrolyte enhances for LIB capacity retention.^[^ ^51^ ^]^ However, a trade‐off between capacity retention and the cell interfacial resistance was found when VC was added to the electrolyte.^[^ ^52^ ^]^ Lim et al. prepared VC microcapsules successfully by solvent exchange, which allows VC to diffuse through the microcapsule shell at an elevated temperature.^[^ ^53^ ^]^ They found that the release of VC timely from microcapsules in LIBs promotes stable SEI layer formation.

Note that the main difference between the microcapsule materials mentioned in this work and core–shell structure battery materials is that the microcapsule shell will be destroyed when the cell fails, and its function is mainly to release the core material to avoid accidents, such as fire, or realize the healing of specific functions of the cell, such as electrical conductivity; however, the shell of the core–shell material will not be damaged (i.e., there is no releasing process), and its function is to improve certain defects of core materials faced by Si‐based LIBs, such as large volume expansion of Si materials and the unstable SEI layers.^[^ ^54^ ^]^

In summary, capsule technology has broad application prospects in the battery field. In addition to prolonging cycling life and improving reliability it can also be used to improve battery safety. In the future, self‐healing separators to avoid internal short circuit and self‐supplementing of Li^+^ to improve circulation capacity are expected through capsule technology for advanced LIBs.

## 8 Conclusion and Outlook

Large‐scale promotion of EVs is one of the main ways to reduce carbon emissions. However, if the safety of power batteries cannot be improved, EVs will be difficult to promote. In addition, power batteries need better electrochemical performance to meet the requirements of fast charging, high mileage, and long life. Designing a high‐safety LIB can solve the LIB fire problem. However, high‐safety often means low electrochemical performance. To achieve cells with both high‐safety and high‐electrochemical performance, a unique method was proposed, that is, cells with built‐in capsules, capsules with built‐in TRRs as explored in this review. This method has great application prospects and engineering value, demonstrated through material preparation, performance testing, and effect verification. However, different capsule types, with different TRRs or different capsule shell materials, have different effects on battery safety and electrochemical performance. In addition, the amount of TRR capsules has a great impact. Overall, the comprehensive performance of TRR capsule‐based batteries is satisfactory. When a reasonable capsule solution is adopted, such as an integrated capsule‐based on CCs, not only better safety and electrochemical performance can be obtained, but also higher specific energy can be obtained. We believe that more efficient methods remain to be discovered. Here, we list several possible directions for future battery safety research.

Advanced batteries based on TRR capsules are in urgent need of research. The LIB technology that has been applied in engineering is developing toward advanced battery technology, such as high‐nickel, large‐capacity, etc., to increase the cell specific energy and reduce the cost. However, high‐nickel means that battery materials are more active and safety issues will become more prominent. Large‐capacity batteries contain more energy, and once the battery is thermally out of control, its harm will be greater. Therefore, TRR capsule technology is needed to realize the advantages of these advanced battery technologies while ensuring their safety.

The optimal design of TRR capsules is very important. In addition to temperature control, the capsule release strategy can also be controlled by TR gases. It is a plausible strategy to construct smart nanopores with controlled wettability in the shells to control eruption gases, which has been employed in mediating ion transport.^[^ ^55^, ^56^, ^57^, ^58^ ^]^ Besides, nature has provided guidance to design shells with nanopores responsive to gases. For example, stomata on the surfaces of leaves open and close to mediate carbon dioxide (CO_2_) uptake and water loss under the action of abscisic acid and CO_2_.^[^ ^59^, ^60^ ^]^ By mimicking the unique working mechanism of stomata, we can design bioinspired retardant capsules with highly sensitive nanopores in the shells, which can transform between open and closed states. Thus, it is meaningful to construct bioinspired retardant capsules with surface nanopores that enhance battery safety.

TRR capsules used in power batteries need to further expand TRR types to obtain better TR suppression. It is necessary to further reduce the TRR amount to avoid affecting the cell specific energy. TRR capsule technology based on fire extinguishing agents is expected to be applied to LIBs. At present, fire extinguishing agents are mainly sprayed and extinguished through on‐board fire extinguishing systems when the battery catches fire. However, a fire extinguishing system independent of the battery increases the complexity of the EV and reduces the total efficiency of the power system, and the cost also needs to be considered. Fire extinguishing agents have a strong extinguishing effect, such as perfluorohexanone (Novec 1230), which is suitable for battery fires.^[^ ^61^ ^]^ Encapsulating it and placing it inside the cell is expected to greatly improve cell safety, increase power system efficiency, and reduce the complexity and cost of EVs.

Integrated capsule technology will become a research hotspot. Integrated capsules do not change the original structure of the cell, so it will not affect existing LIB production lines too much. Integrated capsule technology can make separators, current collectors, etc., hollow to fill them with TRRs. If a suitable material such as PI is selected, not only the safety of the battery can be improved, but also its specific energy can be increased. This advantage is rare in many battery technologies. This technology is expected to be popularized and applied in EVs.

To realize large‐scale applications of capsule technology in power batteries and EVs, it is necessary to further simplify the existing capsule preparation process, improve repeatability, and reduce processing costs. Only in this way can we ensure the application of this technology beyond the research level, so as to truly play a role in promoting EVs and coping with global climate change.

In short, capsule‐based batteries are expected to solve LIB safety problems. Capsules are also expected to achieve self‐extinguishing, self‐healing, and Li^+^ self‐supplementing, to be applied in the EV field. Along with sensing technology,^[^ ^62^ ^]^ it will become part of battery smart functions to provide technical support for safer and longer‐lived LIBs that reduce carbon emissions. Therefore, we suggest that more scientific researchers and companies pay attention to this research field and carry out research and verification work on large‐capacity high‐nickel batteries with capsules.

## Conflict of Interest

The authors declare no conflict of interest.
